# Omaveloxolone promotes functional recovery of spinal cord injury by reducing inflammatory response and regulating macrophage polarization

**DOI:** 10.3389/fnmol.2025.1737798

**Published:** 2026-01-12

**Authors:** Pengtian Zhao, Wenlu Yuan, Jiayi Zhang, Erke Gao, Dejing Zhang, Zhuolin Wu, Yue Zhang, Junbo Chen, Dunxu Hu, Baoyou Fan, Junjin Li, Wenchao Dai, Zhijian Wei, Tao Zhang

**Affiliations:** 1 Department of Orthopedics, Tianjin First Center Hospital, Tianjin Medical University, Tianjin, China; 2 Department of Orthopedics, The Second Qilu Hospital of Shandong University, Jinan, Shandong, China; 3 Department of Orthopedics, National Spinal Cord Injury International Cooperation Base, Tianjin Key Laboratory of Spine and Spinal Cord Injury, Tianjin Medical University General Hospital, Tianjin, China; 4 College of Life Science, Nankai University, Tianjin, China; 5 Department of Spine Surgery, Shengli Oilfield Central Hospital, Dongying, Shandong, China; 6 Department of Neurosurgery, Tianjin Medical University General Hospital, Tianjin, China; 7 Department of Pain Management, The Second Qilu Hospital of Shandong University, Jinan, Shandong, China; 8 Department of Orthopedics, Tianjin Medical University General Hospital, Tianjin, China; 9 Department of Orthopedics, Qilu Hospital of Shandong University, Shandong University Centre for Orthopedics, Advanced Medical Research Institute, Shandong University, Jinan, Shandong, China

**Keywords:** acute inflammation, macrophage, neuroimmunology, Omaveloxolone, spinal cord injury

## Abstract

**Background:**

Spinal Cord Injury (SCI) is a severe central nervous system disorder that initiates inflammatory reactions, exacerbating tissue damage and impeding neuronal repair. Macrophage polarization plays a critical role in this pathological process: it significantly regulates inflammation resolution and tissue regeneration, rendering its modulation a key strategy for SCI repair. Omaveloxolone (Omav), a novel Nrf2 activator, has demonstrated potential in regulating inflammatory responses, suggesting it may serve as a promising candidate for SCI intervention.

**Methods:**

To evaluate the efficacy and underlying mechanism of Omav in SCI repair, a spinal cord contusion model was established in animal subjects. Additionally, an *in vitro* lipopolysaccharide (LPS)-induced macrophage polarization model was constructed to further validate Omav’s effects on macrophage phenotypes. RNA sequencing (RNA-seq) was employed to elucidate the molecular pathways through which Omav modulates post-SCI pathophysiology.

**Results:**

*In vivo* experiments revealed that Omav effectively restored motor function in SCI-induced animals. RNA-seq analysis further demonstrated that Omav reshaped inflammatory cascades following SCI, with a significant impact on macrophage polarization dynamics. Specifically, Omav promoted the formation of an M2-dominant macrophage landscape (a phenotype associated with anti-inflammation and tissue repair) while reducing the pro-inflammatory M1 macrophage phenotype. These findings were corroborated by in vitro studies, which confirmed that Omav directly facilitated M2-type macrophage polarization.

**Conclusion:**

Our results collectively confirm the efficacy of Omav in repairing spinal cord injury by targeting macrophage polarization and regulating inflammatory responses. This study not only highlights the therapeutic potential of Omav for SCI but also provides a novel pharmacological strategy for SCI treatment.

## 1 Introduction

Spinal Cord Injury (SCI), a debilitating neurological affliction, triggers extensive motor, sensory, and autonomic nervous system compromise (Anjum et al., 2020; Hou and Rabchevsky, 2014; Ahuja et al., 2017), culminating in reduced patient quality of life and burdening societies and families economically and psychologically (Courtine and Sofroniew, 2019; Shokur et al., 2018). Epidemiological investigations by the World Health Organization (WHO) and researchers from several Western nations estimate the global SCI incidence rate as averaging 10–40 cases per million individuals, albeit with notable regional variance (García-Altés et al., 2012; Polinder et al., 2007; Thompson et al., 2015). Both China and the United States grapple with relatively high SCI incidence rates, exceeding 40 annual cases per million populace (Quadri et al., 2020). The central nervous system’s (CNS) intrinsic repair capabilities remain constrained, which causes many patients to experience motor dysfunction and sensory impairment (Afshari et al., 2009; Tedeschi and Bradke, 2017). Currently available clinical treatments for SCI, including pharmacological, surgical, and rehabilitative therapies, have not been able to achieve effective recovery of motor and sensory neurological functions (Chen and Levi, 2017; Badhiwala et al., 2019). Therefore, there is an urgent need to find new treatment strategies to promote neurological recovery and improve patient outcomes.

After SCI, neuroinflammation and macrophage polarization play a crucial role in neural repair (Fan et al., 2020; Li et al., 2024; Zhu et al., 2023). The activation of macrophages often exacerbates the inflammatory response, thereby affecting the recovery process (Fu et al., 2022; Hamidzadeh et al., 2017). Hence, unraveling macrophage polarization regulatory pathways emerges as a crucial pivot for post-SCI therapeutic interventions. Current research indicates that after SCI, macrophage polarization is mainly divided into M1 and M2 types (Gensel et al., 2011; Zhou et al., 2020). M1 macrophages characteristically drive pro-inflammatory responses, whereas M2 macrophages chiefly regulate anti-inflammatory effects and tissue regeneration. A mounting body of evidence uncovers the potential of M1 macrophages to upregulate destructive inflammatory cytokines, exemplified by IL-1β and TNF-α, thereby exacerbating host cell damage and hindering tissue repair (Fuchs et al., 2016; Locati et al., 2020). In contrast, M2 macrophages exert anti-inflammatory and tissue repair functions by clearing necrotic tissue debris and releasing protective factors, including IL-4 and IL-10 (Kigerl et al., 2009; Ma et al., 2015; Gensel et al., 2011; Zhou et al., 2020).

Therefore, mounting evidence advocates the strategic modulation of macrophage behavior as a therapeutic avenue for SCI, encompassing macrophage recruitment and proliferation inhibition (Kroner et al., 2014; Gensel and Zhang, 2015), M1 phenotype activation pathway suppression, and promoting M2 macrophage transition (Gao et al., 2021; Wang et al., 2020; Lin et al., 2024). While prior studies have illuminated macrophage roles in SCI, examination of Omaveloxolone (Omav) impacts on macrophage polarization and regulatory mechanisms remains comparatively scarce. Delving into Omav’s influences on macrophage polarization and its regulatory underpinnings thus holds substantial scientific and clinical relevance.

Omav is a novel Nrf2 activator and a synthetic oleanane triterpenoid currently under clinical evaluation for treating Friedreich’s ataxia (FA) (Lee, 2023; Reisman et al., 2019). In recent years, the Nrf2 conduit has commanded burgeoning scholarly notice. It occupies a central station in tempering inflammatory cascades and advancing neural restitution through its dominion over bioenergetic metabolism. Prior studies have illuminated that provocation of the Nrf2 apparatus can recalibrate both inflammation and oxidative perturbations, thereby mollifying the pathological manifestations allied with SCI (Zhou et al., 2020; Fuchs et al., 2016; Locati et al., 2020; Kigerl et al., 2009). Omav evinces antioxidative and anti-inflammatory proclivities, while likewise modulating mitochondrial energetic orchestration (Ma et al., 2015; Kroner et al., 2014). In addition, antecedent explorations have disclosed that Omav amplifies Nrf2 expression in dermal maladies, subsequently heightening the abundance of NQO1 and HO-1 (Gensel and Zhang, 2015).

Additionally, Omav reduced the content of IL-1β, promoted the phosphorylation of NF-κB, and activated the expression of Nrf2 target genes in rat astrocytes. These effects diminished the expression of MMP-9, indicating Omav’s potential benefits for treating neurodegenerative diseases (Yang et al., 2019). Furthermore, experimental research has confirmed that Omav has the potential to regulate inflammatory responses and promote neural repair (Jian et al., 2022; Jiang et al., 2022).

The objective of the present inquiry is to elucidate the mechanism whereby Omav orchestrates macrophage M1/M2 polarization in the aftermath of SCI, employing RNA sequencing analysis as the principal instrument of interrogation. We used Bulk RNA-seq and RNA-seq of flow-sorted macrophages, and combined differential expression gene (DEG) screening, gene ontology (GO) and KEGG pathway enrichment, PROGENy tools, CIBERSORT algorithm, and other bioinformatics methods to systematically evaluate Omav’s impacts on macrophage M1/M2 polarization and their functional ramifications following SCI are warranted. Such exhaustive analyses can illuminate Omav’s molecular underpinnings in modulating macrophage polarization and neuroinflammation. By unraveling these mechanisms, we aspire to inform innovative therapeutic interventions that improve prognosis and life quality for individuals grappling with SCI.

## 2 Materials and methods

### 2.1 SCI model

This study used female C57BL/6JNifdc mice (18–22 g, 6–8 weeks). The mice were purchased from Beijing Huafukang Biotechnology Co., Ltd. (Beijing, China, License No.: SCXK (Jing) 2024-0003) and were inhabited a temperature-controlled environment maintained between 22 and 24 °C and relative humidity levels spanning 60–80%, subjects benefited from unrestricted sustenance and hydration access amidst a 12-h photoperiod schedule. The animal study was approved by the Animal Welfare and Ethics Committee of Tianjin Medical University General Hospital (Approval No.: IRB2022-DWFL-201). All surgical procedures were performed in a sterile environment. The mouse T10 spinal cord contusion model was established using a modified Allen method adapted from rat spinal cord contusion models (Hua et al., 2024). Mice were subjected to isoflurane-induced anesthesia, succeeded by a longitudinal incision penetrating the skin and fascia, centered upon the T10 level. Muscular retraction then exposed the T9–T10 spinous processes, with the T10 lamina’s removal divulging the underlying spinal cord. The T9 and T10 spinous processes found stabilization via a spinous process fixator, setting the stage for spinal cord contusion induction utilizing the NYU Impactor-III. The Omav intervention arm received 100 μL (10 mg/kg) Omav intraperitoneally, whereas controls received a PBS volume equivalent. Omav administration commenced half an hour post-SCI establishment and extended daily for a seven-day regimen, with dosage calibration derived from preceding investigations (Jiang et al., 2022; Creelan et al., 2017; Liu et al., 2016).

### 2.2 Cell culture

Extraction of bone marrow from the femur and tibia of male C57BL/6 mice inaugurates the experimental protocol. Subsequent incubation of these cells in an induction medium containing DMEM/F-12, FBS, penicillin/streptomycin, and M-CSF facilitates their differentiation into M0 macrophages over 7 days, establishing a control group baseline. Further exposing M0 macrophages to LPS elicits a pro-inflammatory response, henceforth referred to as the LPS group. Conversely, an LPS and Omav co-treatment regimen for 24 h modulates macrophage polarization, instigating an anti-inflammatory cascade; these cells are designated as the SCI plus Omav group.

### 2.3 Basso mouse scale (BMS) score

Utilizing BMS facilitates the evaluation of hind limb motor function restoration in murine subjects subsequent to SCI. Specifically, two trained experimenters conduct a 4-min evaluation of the mice in an empty room. Scores range from 0, indicating no motor function, to 9, representing normal function (Basso et al., 2006).

### 2.4 Motor-evoked potential (MEP)

Eight weeks after SCI, electrophysiological activity of the hind limbs of mice was assessed using an electrophysiological device (Ran et al., 2023). Under the cloak of anesthesia, mice were prepped for electrophysiological assessment, commencing with strategic placement of two stimulating electrodes—one situated posteriorly on the cranium and the other aligned with the cervical region to document and juxtapose the peak amplitude and latency of MEPs across diverse murine cohorts.

### 2.5 Catwalk gait analysis

Eight weeks after SCI surgery, the CatWalk XT system, comprising both hardware and software components, To objectively dissect the paw imprints, motor functionality, and interlimb synchrony of mice (Hua et al., 2024; Creelan et al., 2017), we harnessed the CatWalk gait analysis system. Bathed in the seclusion of a dark and quiet ambiance, mice embarked on a training regimen, traversing the illuminated glass corridor at least thrice. Once acclimatized, high-speed camera surveillance chronicled each unique paw impression as mice ambulated across the walkway. Data collection and analysis were performed using the CatWalk XT software, including calculation of the regularity index (RI) and average speed. The RI, defined as the ratio as a ratio of standard step sequence instances to aggregate paw placements, as gait analysis in SCI unveils, a direct correlation emerges between accurate gait sequences and regularity index escalation during movement. Additionally, stride length, a pivotal gait parameter, frequently succumbs to a marked decline post-SCI.

### 2.6 Tissue immunofluorescence staining

Seven days after SCI, mice were deeply anesthetized with isoflurane, and the heart was perfused with phosphate-buffered saline (PBS). Once the liver appeared pale, perfusion was continued with 4% paraformaldehyde to fix the tissues. The spinal cord tissue was then dissected and fixed in 4% paraformaldehyde for 1 day. The tissue was subsequently dehydrated by sequential immersion in 10, 20, and 30% sucrose solutions, each for 1 day. Following desiccation, the spinal cord parenchyma was enshrouded in OCT compound (SAKURA, 4583) and sequestered in a frozen state at −20 °C. Employing a cryostat (CM30505; Leica), the tissue was sculpted into cryosections of 10 μm breadth. The slices, once stabilized with 4% paraformaldehyde for 30 min, were rinsed in PBS and thereafter subjected to a 1-h blockade and permeabilization using 5% fetal bovine serum (FBS) with 0.25% Triton X-100. Subsequently, the preparations were co-incubated with primary antibodies targeting CD68 (1:300, Abcam, ab53444), iNOS (1:200, Abcam, ab178945), and CD206 (1:200, Abcam, ab64693), Iba1 (1:200, ab283319) and afterward revealed with their cognate secondary antibodies. Chromatic delineation of nuclei was achieved with DAPI for 10 min, and imagery was acquired through a Leica fluorescence microscope (Leica TL LED, Germany). Quantitative scrutiny ensued via ImageJ software.

### 2.7 Cellular immunofluorescence staining

Immerse cells in a 4% PFA solution for fixation, succeeded by a blocking solution of 10% goat serum and 0.5% Triton X-100 for 1 h at ambient temperature. Subsequent to this, subject cells to a prolonged 4 °C incubation with primary antibodies CD68, iNOS, and CD206. Following this incubation period, introduce corresponding secondary antibodies for an additional hour at room temperature. Finally, stain nuclei with DAPI for 10 min, capture images using a Leica fluorescence microscope (Leica TL LED, Germany), and perform quantitative analysis using ImageJ software.

### 2.8 HE staining

After preparing the paraffin sections, incubate the sections at 60 °C within the unwavering climes of an oven for a four-hour interval precedes a successive immersion in xylene I and xylene II, each enduring for a 10-min span each. Next, immerse the sections in ethanol solutions at different concentrations (100, 95, 90, 80, and 70%) for 5 min each. Rinse the sections with distilled water for 1 min, stain with hematoxylin (Solarbio, #G1120, Beijing, China) for 2 min, and differentiate using 1% hydrochloric acid solution. Immerse sections in distilled water for a 2-min rinse, then apply a 0.5% eosin solution for staining. Execute a sequential dehydration protocol, transitioning sections through 70, 80, 90%, and ultimately 100% ethanol solutions. Subsequently, subject slides to 5-min immersions in xylene I and xylene II, culminating in neutral resin encapsulation. Conclude the procedure by capturing high-resolution images using an automated tissue *in situ* multi-label quantitative analyzer (Vectra Polaris, PerkinElmer).

### 2.9 Flow cytometry

Isolated BMDM cells underwent 70 μm cell filtration, yielding a single-cell suspension primed for incubation with FITC anti-rat CD11b and PE anti-rat CD86 antibodies over 30 min. Succeeding this, a 15-min exposure to cell-fast™ Fix/Perm Buffer Set preceded an additional 30-min incubation with CD206 (mannose receptor) antibody in dimmed, ambient conditions. Thereafter, cells encountered goat anti-mouse IgG H&L for another round of incubation. Concluding this sequence, samples were harvested via BD LSRFortessa flow cytometer and their data underwent FlowJo-mediated analysis.

### 2.10 Western blotting (WB)

Protein quantification transpired via a BCA assay (Thermo Scientific), establishing the foundation for subsequent 12.5% SDS-PAGE-mediated target protein segregation and PVDF membrane transference. Upon PVDF membrane blocking with 5% non-fat milk in TBST, an overnight 4 °C incubation with GAPDH, iNOS, and CD206 antibodies ensued, followed by secondary antibody interaction under optimized parameters. Protein band visualization emerged through ECL reagents, producing images documented with the ChemiDoc XRS system. ImageJ software-directed quantitative analyses provided a comprehensive evaluation of protein expression patterns.

### 2.11 Enzyme-linked immunosorbent assay (ELISA)

On the 3rd day post-SCI, the concentrations of inflammatory cytokines TNF-α, IL-1β, and IL-6 within spinal cord tissue homogenates were ascertained utilizing respective IL-6, IL-1β, and TNF-α ELISA kits. The spinal cord tissue homogenate supernatant was collected and processed to extract various cytokines. Following the kit instructions, the samples were diluted and centrifuged, then biotinylated antibodies from the kit were added to a 96-well plate and incubated at 37 °C for 1 h, followed by washing the plate three times. Then, enzyme conjugate working solution was added, and the plate was incubated at 37 °C for 30 min. A quintet of washes heralded substrate incorporation, succeeded by a 15-min incubation epoch at 37 °C. Culminating in stop solution admixture, the optical density values of individual wells were instantaneously gauged via a microplate reader at a 450 nm wavelength. Each group contained three samples, and each sample was tested three times to ensure the reliability of the experimental results.

### 2.12 RNA-seq data processing and alignment

Raw base call (BCL) files generated on the Illumina NovaSeq 6000 platform were converted to demultiplexed FASTQ files according to sample-specific barcodes. Adapter removal and quality filtering were performed using fastp (v0.23.4), which trimmed residual adapter sequences and discarded reads containing low-quality bases or excessive ambiguous nucleotides (N), yielding high-quality clean reads. These clean reads were subsequently aligned to the mouse reference genome (*Mus musculus*, GRCm39) using HISAT2 (v2.2.1). Gene models were defined according to the Ensembl *Mus musculus* gene annotation (Ensembl release 104, GTF format). For each sample, gene- and transcript-level expression was quantified with StringTie (v2.2.3) and reported as fragments per kilobase of transcript per million mapped reads (FPKM), and a corresponding gene-level raw count matrix was generated for downstream statistical analysis in R software.

### 2.13 Normalization, filtering, and principal component analysis

The raw count matrix was imported into DESeq2 (v1.40) for normalization and differential expression analysis. To reduce noise from extremely lowly expressed genes, we first removed genes with fewer than 10 raw counts in at least two samples, resulting in 15,680 genes retained for downstream analyses. Library size normalization was performed using the median-of-ratios method implemented in DESeq2, and gene-wise dispersion parameters were estimated under the negative binomial framework. Variance-stabilizing transformation (VST) was then applied to the normalized counts to obtain an expression matrix with approximately homoscedastic variance across the dynamic range.

Principal component analysis (PCA) was conducted on the centered VST matrix using the base R function prcomp. The first two principal components were visualized with ggplot2 (v3.4) as a scatterplot, and 95% confidence ellipses were overlaid to illustrate within-group variability. For differential expression testing, a simplified design formula ~ condition was used in DESeq2 to fit negative binomial generalized linear models. Wald statistics were calculated for group contrasts, and log₂ fold changes (log₂FC) were shrunk using the apeglm method to improve effect-size estimation, particularly for lowly expressed genes. Genes with an adjusted *p* value (Benjamini–Hochberg–corrected, *p*_adj_) < 0.05 and an absolute log₂FC > 2 were considered differentially expressed (DEGs). Volcano plots were generated with ggplot2, and clustered heatmaps were produced using pheatmap (v1.0.12) based on the top 140 DEGs, with rows centered to highlight relative expression changes; red and blue side bars indicated upregulated and downregulated genes, respectively.

### 2.14 Gene set enrichment analysis (GSEA)

Enrichment of significant DEGs was performed using clusterProfiler v4.6. GO-BP: enrichGO (*q* < 0.05, BH correction); redundancy was removed using simplify. Subsequently, a network was constructed using GOSemSim v2.23, igraph v1.5, and ggraph v2.1, based on Wang semantic similarity >0.3, with node size ∝ − log₁₀(*p*). KEGG: enrichKEGG (*p* < 0.05); displayed as a bubble chart, where bubble size represents the number of genes and color indicates −log₁₀(*p*_adj_). GSEA: all genes were ranked by log₂FC; fgsea (multilevel mode) was used to run MSigDB C2 (mouse homolog). Pathways with |NES| > 1.5 and *p*_adj_ < 0.05 were considered significant and presented as a ridgeline-dot combination plot (ridgeline shows gene distribution, dot-plot encodes NES size/direction).

### 2.15 Pathway activity inference (PROGENy)

We calculated the Z-scores of 11 classic pathways, including MAPK, PI3K, and NF-κB, using the PROGENy model implemented in decoupleR v2.0. The analysis was performed with the top 1,000 genes, specifying the organism as mouse, and *Z*-scores were computed. The resulting data were visualized as a heatmap using ComplexHeatmap; hierarchical clustering was conducted using complete linkage criteria based on Euclidean distance.

### 2.16 Immune cell deconvolution (CIBERSORT)

First, we used biomaRt (Ensembl 104) to convert mouse gene symbols to human HGNC uppercase symbols. Next, we identified the intersection with the LM22 gene signature matrix, which contains at least 510 genes. We then called CIBERSORT.R with the LM22 gene signature matrix, performing 1,000 permutations and disabling quantile normalization, to estimate the relative proportions of immune cell types. We retained 10 cell types with abundance greater than 0.5% and plotted a heatmap normalized by rows, along with a stacked bar chart. For inter-group comparisons, we used a two-tailed unpaired *t*-test with Benjamini–Hochberg multiple testing correction.

### 2.17 Statistical analysis

Statistical scrutiny transpired via GraphPad Prism 9.2.0 software (GraphPad software, San Diego, CA, USA). Student’s *t*-test in concert with one-way analysis of variance (ANOVA), interlaced with Tukey’s *post hoc* assay, constituted the substratum for between-group evaluations, with the demarcations of significance ordained as follows: *p* < 0.05, *p < 0.01, **p < 0.001, ***p < 0.0001. Each experimental endeavor underwent at least three replications to ensure robustness. Subsequently, results manifested as the standard error of the mean (SEM).

## 3 Results

### 3.1 Recovery of functional performance in mice after SCI treated with Omav

**Figure 1:**
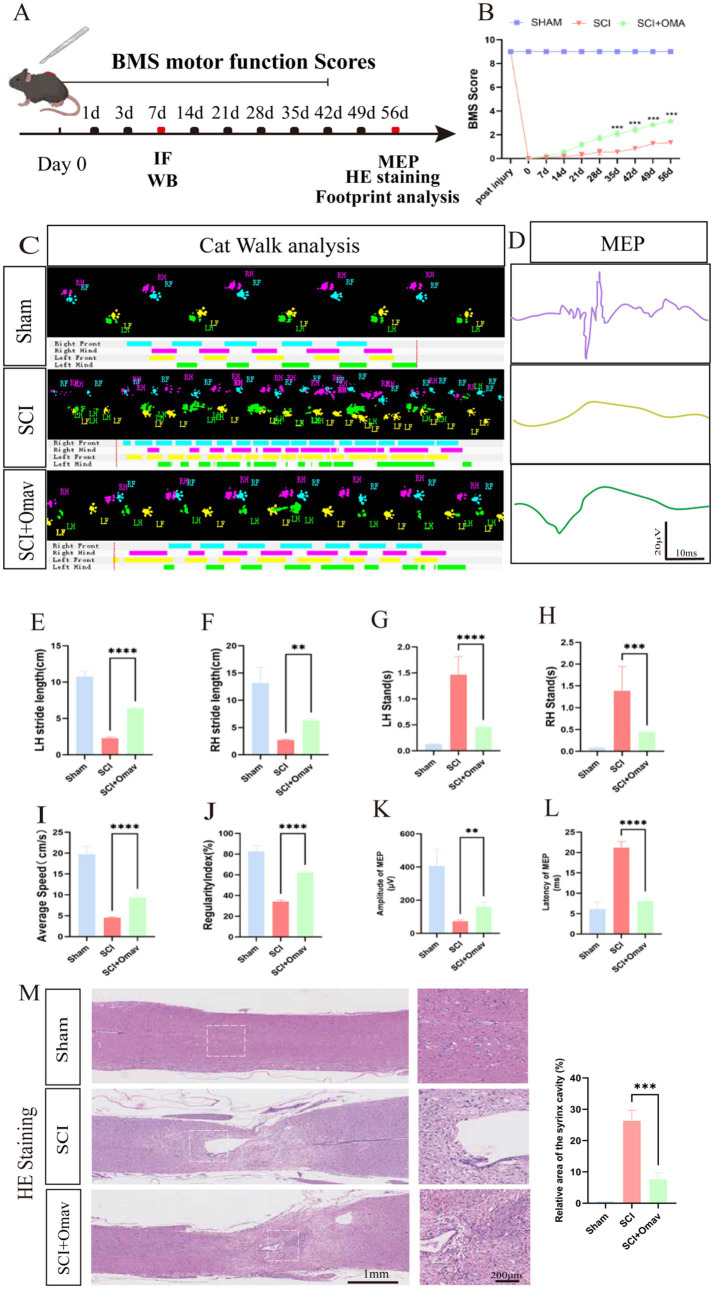
Function recovery of mice by Omav after SCI *in vivo*. **(A)** Schematic flowchart of animal experimentation. **(B)** Temporal evolution of BMS scores post-SCI revealed significant improvement in mice receiving Omav treatment (*n* = 6). **(C)** Exemplary paw step visuals and limb support timing plots from CatWalk gait analysis. **(D)** Electrophysiological appraisals via MEP analysis at week 8 post-injury underscored enhanced neural signal transmission in Omav-treated mice. **(E–J)** Comprehensive CatWalk metrics at week 8 post-injury, encompassing mean velocity, regularity index, and hindlimb stance characteristics (*n* = 6, RH: right hindlimb, LH: left hindlimb). **(K–L)** Analysis of MEP was performed as an electrophysiological assessment in both groups at week 8 post-injury (*n* = 6). **(M)** H&E staining showcased reduced spinal cord lesion dimensions following Omav administration and relative area of the syrinx cavity (*n* = 3, One-way ANOVA, ***p < 0.001). Data expressed as mean ± SEM. Statistical scrutiny via One-way ANOVA unveiled significant results: *p < 0.05, **p < 0.01, ***p < 0.001.

Employing BMS scores as a metric of motor function recuperation post-SCI in mice, we discovered that Omav intervention corresponded with significantly enhanced BMS scores from 21 days post-injury (dpi) onwards, compared to untreated SCI controls (Figure 1B). Cat Walk gait analysis further elucidated superior hind limb coordination among SCI mice receiving Omav therapy (Figures 1C,E–J). To appraise nerve conduction enhancement, electrophysiological assessments were conducted on the subjects. MEP data unveiled shortened latency and amplified amplitude in SCI mice administered Omav (Figures 1D,K–L), suggesting improved neural signal transmission. Histological examinations through H&E staining illuminated reduced lesion dimensions in Omav-treated SCI mice (Figure 1M). Collectively, these findings corroborate Omav’s potential in fostering functional restoration following SCI. To investigate the long-term effects of Omav on functional recovery after SCI, we established three experimental groups: sham, SCI, SCI+Omav. The recovery of mice was monitored over an 8-week period post-injury (Figure 1A).

### 3.2 Significant changes in the overall transcriptome level of SCI tissues after Omav treatment

**Figure 2:**
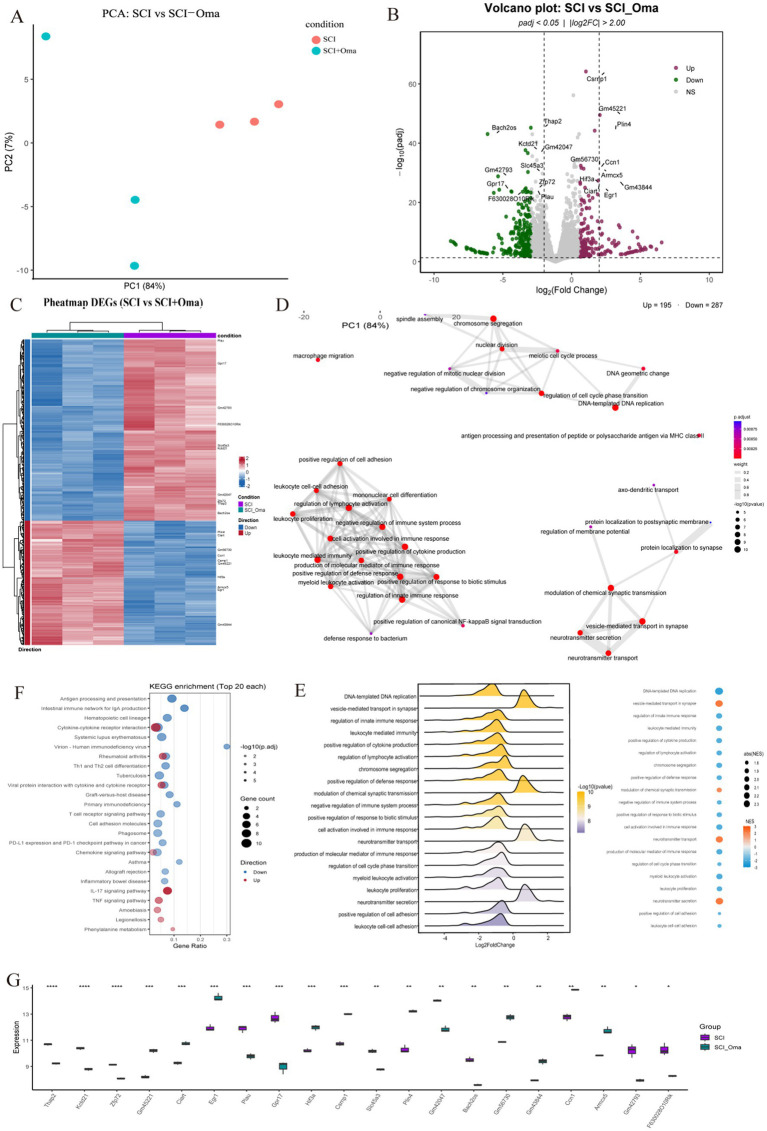
Transcriptome analysis of SCI group and SCI + Omav group. **(A)** Principal-component analysis (PCA): SCI and SCI + Oma samples separate clearly along PC1, indicating that Oma treatment induces a marked transcriptome-wide shift. **(B)** Volcano plot: with thresholds *p*_adj_ < 0.05 and |log₂FC| > 2, 165 genes are up-regulated and 257 down-regulated; representative DEGs are labeled. **(C)** Heat-map of DEGs: A red/blue side bar marks genes that are up- or down-regulated, respectively. **(D)** GO-BP semantic network: significantly enriched biological-process terms are linked by semantic similarity (>0.3). **(E)** GSEA overview: ridgeline plots show log₂FC distributions for 13 significant pathways; the adjacent dot-plot encodes NES (color) and |NES| (size). **(F)** KEGG bubble plot: Bubble size reflects the number of pathway genes; color represents −log10(*p*_adj_), highlighting immune- and redox-related pathways. **(G)** Box-and-jitter plot of key genes: Expression of 20 candidate genes is compared between the two treatments; stars denote t-test significance.

First, principal component analysis (PCA) was performed on the sequencing results of six bulk RNA-seq SCI samples (Figure 2A), revealing that the first principal component (PC1) explained about 84% of the variation in expression.

Further DEGs analysis (Figures 2B,C) identified a total of 422 significant DEGs with a screening threshold of |log₂FC| > 2.0 and FDR < 0.05, including 195 upregulated genes and 287 downregulated genes. Functional analysis revealed that downregulated genes were concentrated in immune-related transcription regulation (e.g., Bach2os/Bach2) and inflammation-induced apoptosis (e.g., Gpr17); while upregulated genes were mainly involved in matrix-related proteins, driving angiogenesis, fibroblast adhesion, wound healing (e.g., Ccn1/Cyr61), and tissue repair-related functional modules (e.g., Csrnp1). Heatmap clustering analysis (Figure 2C) showed that upregulated and downregulated genes formed two distinct modules, which could completely differentiate SCI and SCI + Omav group samples under unsupervised conditions.

GO biological process (GO-BP) enrichment network analysis (Figure 2D) and gene set enrichment analysis (GSEA) (Figure 2E) results indicated that Omav significantly inhibited immune response activation, such as myeloid leukocyte activation and macrophage migration, among other inflammation-related pathways. Meanwhile, neurotransmitter transport/secretion and cell adhesion-related pathways showed mild activation. Omav simultaneously suppressed immune-inflammatory pathways and cell cycle pathways, showing that its anti-inflammatory and anti-proliferative effects support synaptic repair. This suggests that Omav treatment inhibits immune-inflammatory pathways and cell cycle pathways, indicating that it’s anti-inflammatory and anti-proliferative effects complement synaptic repair. It also reflects that the spinal cord tissue after Omav treatment may be undergoing a functional state transition from a high metabolic, oxidative stress-active inflammatory state to a tissue repair-oriented state.

KEGG signaling pathway enrichment analysis (Figure 2F) further validated the above results, finding that Omav treatment significantly downregulated antigen processing and presentation functions and chemokine signaling pathways; while significantly upregulating cell adhesion molecules (CAMs), IL-17 signaling, and phenylalanine metabolism signaling pathways. These changes suggest that Omav may exert a dual regulatory role in both anti-inflammation and promoting antioxidant effects.

Box plot analysis of key genes (Top 20) (Figure 2G) further clarified the changes in key genes after Omav treatment: the expression levels of Gpr17, Plau, and Thap2 significantly decreased, suggesting a reduction in expression after drug treatment, which may alleviate inflammation or glial responses. In contrast, Hif3a, Egr1, and Ccn1 significantly increased, indicating that their expression levels rose after drug treatment, closely related to hypoxic responses and injury repair, potentially promoting the repair process.

### 3.3 Analysis of PROGENy signaling pathways and immune cell subpopulation changes after SCI with Omav treatment

**Figure 3:**
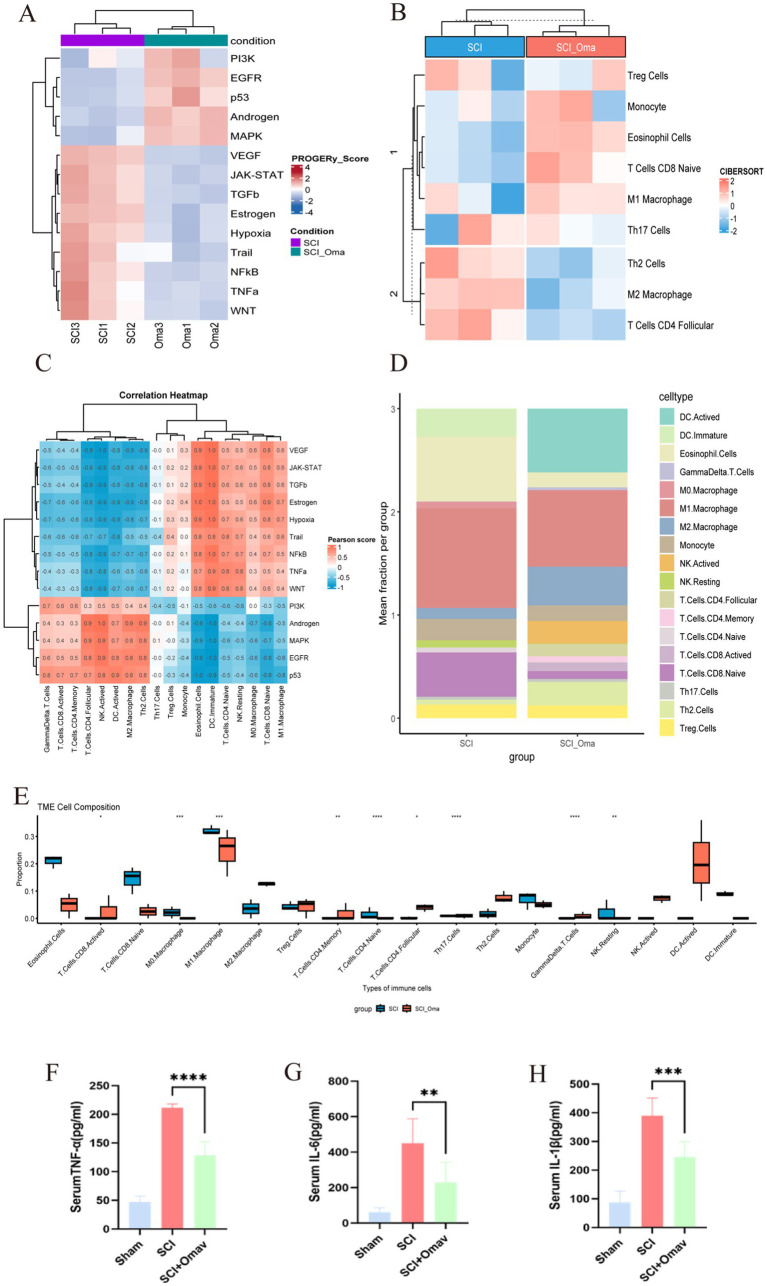
Transcriptome analysis of SCI group and SCI + Omav group. **(A)** PROGENy pathway heat-map: Z-scores for 11 canonical signaling pathways in SCI versus SCI + Oma; red/blue indicate relative activation or inhibition. **(B)** CIBERSORT immune-cell heat-map: Relative abundances of 10 major immune-cell types, hierarchically clustered into two groups. **(C)** Stacked bar chart of immune composition: Mean proportions of immune-cell types in each group; colors correspond to the legend at right. **(D)** Stacked bar chart of immune cell proportions: comparative analysis of mean Immune cell proportions between two groups; colors correspond to cell types in the legend. **(E)** Immune-cell box-and-jitter plots: pairwise comparisons of significantly altered immune-cell fractions; stars mark t-test significance. **(F–H)** Serum TNF-α, IL-1β, and IL-6 (*n* = 6) (*p < 0.05, *** *p* < 0.001).

To gain a deeper understanding of the specific signaling pathways affected by Omav after spinal cord injury, we used the PROGENy tool to assess pathway activity (Figure 3A). The results showed that Omav treatment significantly reduced the activity of MAPK, PI3K, and NF-κB pathways in spinal cord tissue after SCI; while p53 and hypoxia pathways were mildly upregulated.

Subsequently, we analyzed immune cell infiltration using the CIBERSORT algorithm (Figures 3B,C), finding that the proportion of M1 macrophages in SCI spinal cord tissue significantly decreased after Omav treatment, whilst the contingent of M2 macrophages exhibited a marked augmentation; additionally, the proportion of regulatory T cells (Treg) also showed a slight increasing trend. This indicates that the changes in these pathways after Omav treatment are related to macrophage polarization.

Box plot analysis of immune cells (Figures 3D,E) further clarified the changes in immune cell distribution ratios after Omav treatment: the proportion of activated dendritic cells (DC.Actived) and M1 macrophages significantly decreased (*p* < 0.001), suggesting that inflammation in SCI was suppressed after Omav treatment; while M2 macrophages and regulatory T cells (Treg) significantly increased, indicating that Omav treatment can promote the local immune microenvironment to shift toward inflammation suppression and tissue repair. In addition, we tested serum levels of IL-1β, IL-6, and TNF-α using ELISA kits. It revealed that after SCI, the levels of pro-inflammatory cytokines increased, which were decreased after Omav treatment (Figures 3F–H). These trends align with the classical characteristics of macrophages transitioning from pro-inflammatory (M1) to tissue repair (M2). This underscores the necessity to explore the research value of reducing inflammatory response and regulating macrophage polarization after SCI with Omav treatment.

### 3.4 Omav regulates macrophage/microglia polarization toward the M2 phenotype *in vivo*

**Figure 4:**
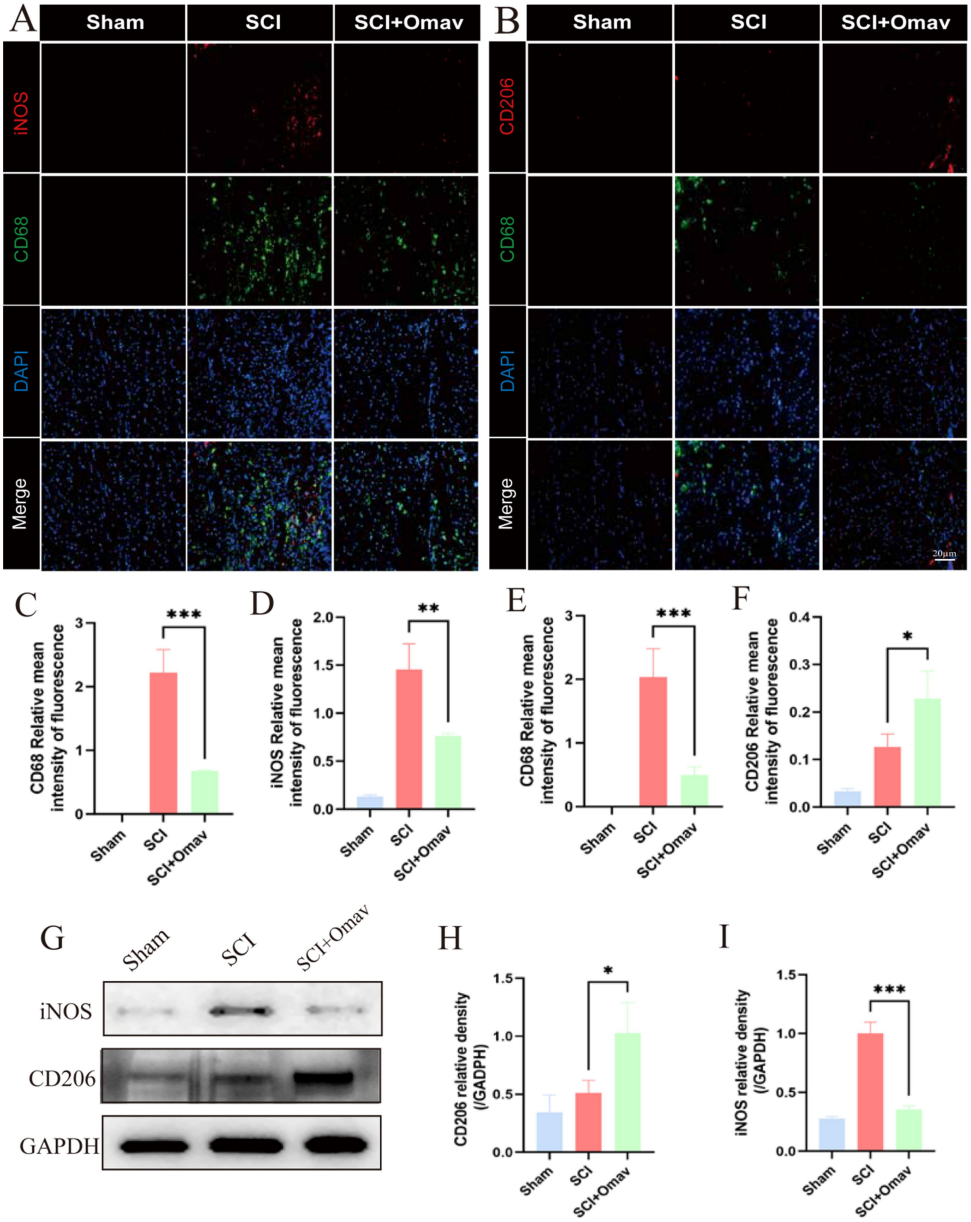
The effect of Omav on the regulation of M1/M2 polarization *in vivo*. **(A)** Exemplary immunofluorescence staining images capture iNOS (red) and CD68 (green) within each group (*n* = 3), accentuated by DAPI nuclear labeling (blue). Scale bar: 20 μm. **(B)** Representative immunofluorescence images showcase CD206 (red) and CD68 (green) staining across all groups (*n* = 3), contrasted against DAPI-highlighted nuclei (blue). Scale bar: 20 μm. **(C,D)** Quantitative analyses unveil the prevalence of CD68+ and iNOS+ cells (*n* = 3). **(E,F)** Similar quantitative appraisals illuminate the abundance of CD68+ and CD206+ cells (*n* = 3). **(G–I)** Protein expression profiles and corresponding quantifications divulge iNOS and CD206 levels amidst diverse experimental groups (*n* = 3).

To explore the mechanism by which Omav repairs spinal cord injury by regulating macrophage polarization, we performed immunofluorescence staining on spinal cord tissue at 7 dpi. We stained for macrophage marker CD68 and M1 phenotype marker iNOS (Figure 4A). Our findings unveiled a substantial decline in CD68 and iNOS positive cell counts (Figures 4C,D). Concomitantly, we harnessed CD68 and CD206 immunolabeling to illuminate macrophage/populations and M2 phenotypes (Figure 4B). Subsequent analyses unveiled diminished CD68+ cell presence in SCI mice receiving Omav (Figure 4E), while CD206+ cell proportion experienced a notable upswing (Figure 4F). In corroboration, western blot assays echoed this mechanistic shift (Figure 4G). Findings highlighted a significant elevation in CD206 levels among the SCI animals treated with Omav cohort compared to controls (Figure 4H), alongside a marked reduction in iNOS expression levels (Figure 4I).

**Figure 5:**
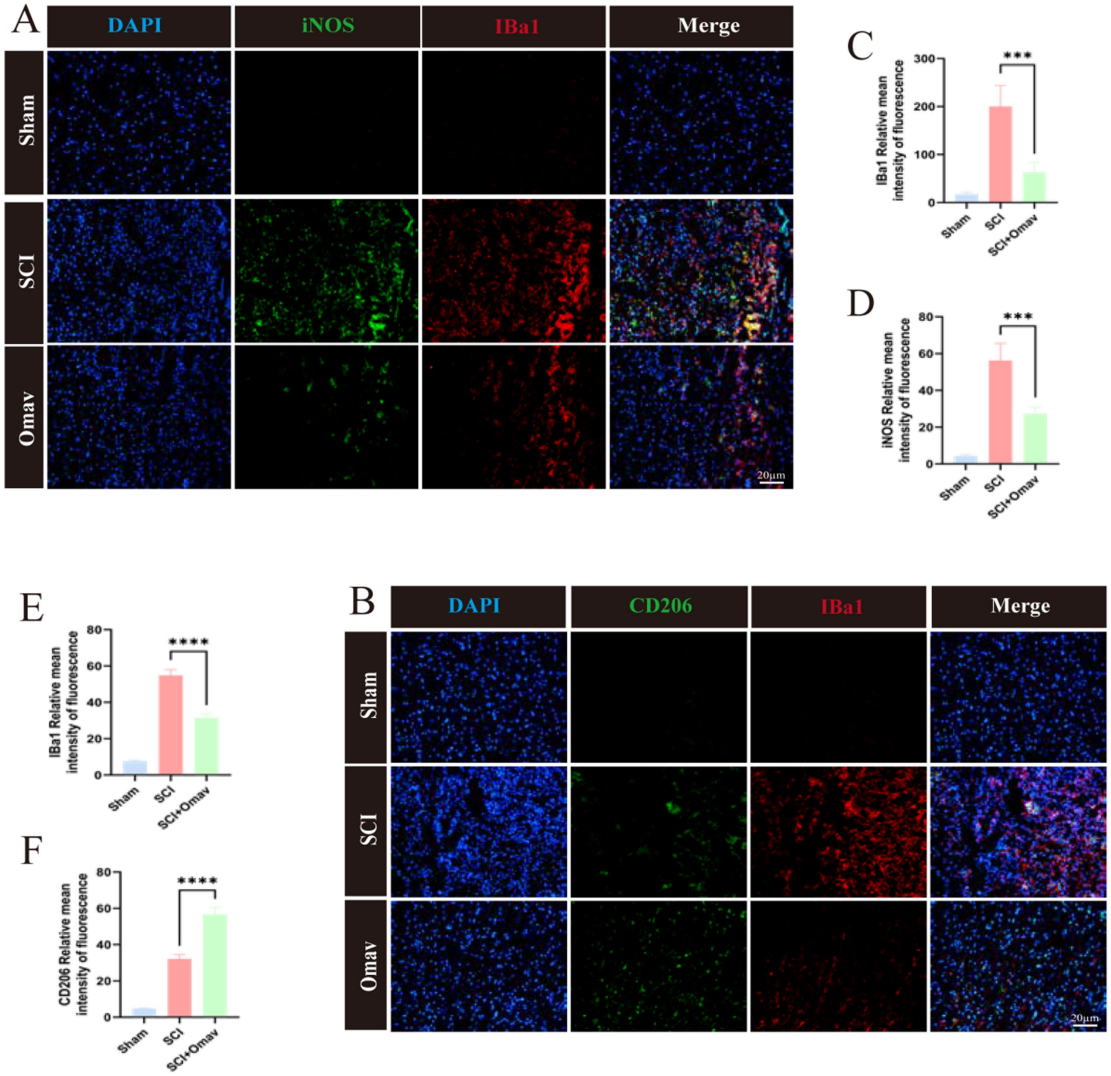
The effect of Omav on the regulation of M1/M2 polarization *in vivo*. **(A)** Exemplary immunofluorescence staining images capture iNOS (green) and Iba1 (red) within each group (*n* = 3), accentuated by DAPI nuclear labeling (blue). Scale bar: 20 μm. **(B)** Representative immunofluorescence images showcase CD206 (green) and CD68 (red) staining across all groups (*n* = 3), contrasted against DAPI-highlighted nuclei (blue). Scale bar: 20 μm. **(C,D)** Quantitative analyses unveil the prevalence of Iba1+ and iNOS+ cells (*n* = 3). **(E,F)** Similar quantitative appraisals illuminate the abundance of Iba1+ and CD206+ cells (*n* = 3).

Subsequently, we stained for microglia marker Iba1 and M1 phenotype marker iNOS (Figure 5A). The results showed a significant reduction of Iba1 and iNOS positive cells (Figures 5C,D). On the other hand, we stained for microglia marker Iba1 and M2 phenotype marker CD206 (Figure 5B). The results demonstrated a significant decrease in Iba1+ cells in the SCI + Omav group (Figure 4E), while the proportion of CD206+ cells significantly increased (Figure 5F).

Collectively, these results evince Omav’s capacity to modulate macrophage/microglia polarization in favor of the neuroprotective M2 phenotype *in vivo*.

### 3.5 Acquisition process of BMDMs and the regulatory effects of Omav on LPS-induced M1/M2 polarization in BMDMs

**Figure 6:**
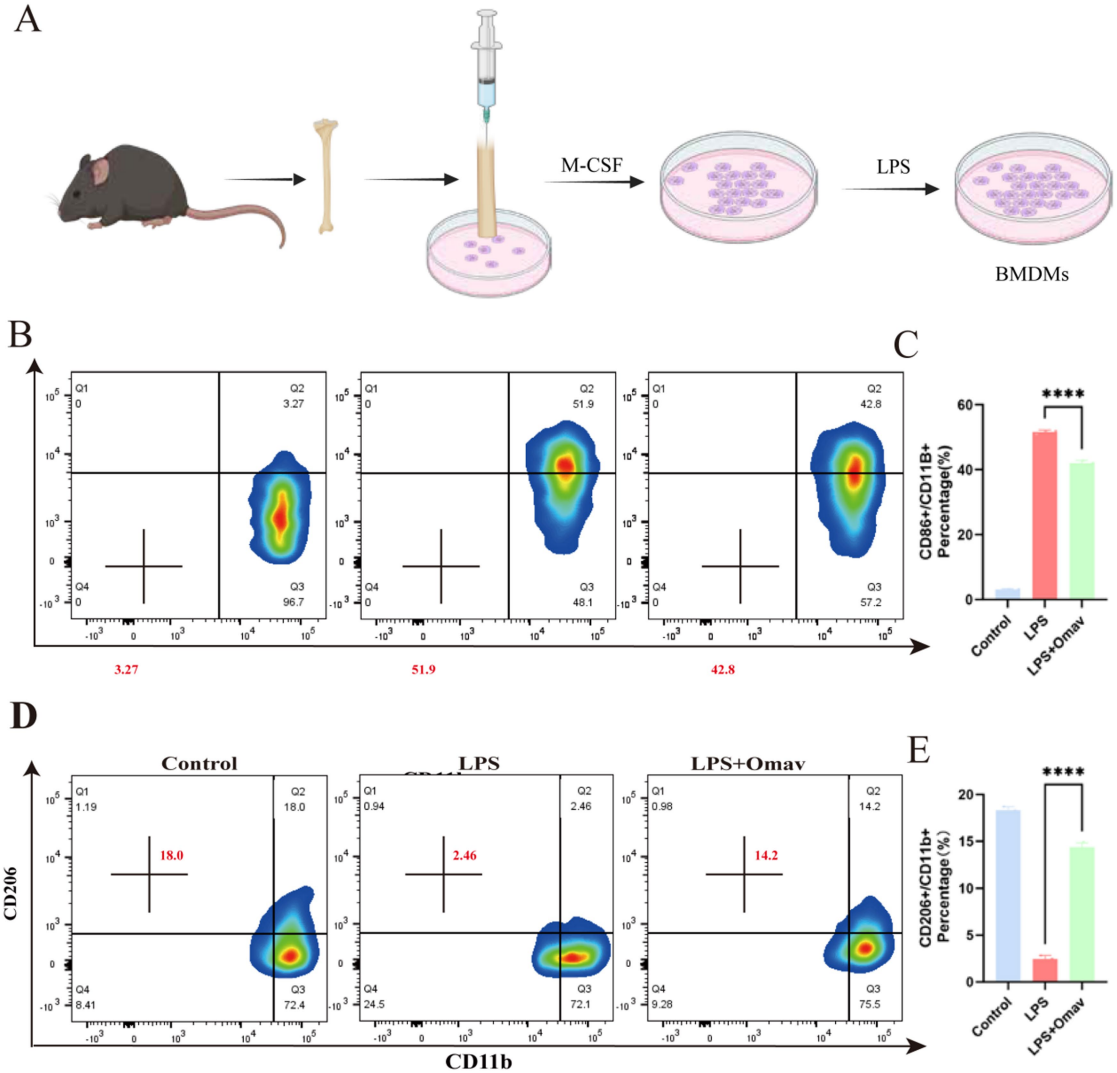
The regulation of Omav on M1/M2 polarization in LPS-stimulated BMDMs. **(A)** Schematic diagram of BMDMs extracted and induced from mice femur. **(B,C)** Flow cytometry assays illuminated a dampened M1 polarization, as evidenced by reduced CD86+ M1 phenotype prevalence in Omav-treated, LPS-stimulated BMDMs (*n* = 3). **(D,E)** Conversely, a CD206+ M2 phenotype surge following Omav administration unveils its potential to tilt the balance toward an M2-dominant profile in the same BMDM population. Statistical analyses via One-way ANOVA establish significant findings: *p < 0.05, **p < 0.01, ***p < 0.001. Together, these data indicate Omav’s capacity to orchestrate macrophage polarization dynamics in favor of an anti-inflammatory milieu.

Primary BMDMs were extracted from adult male C57 mice (Figure 6A). An LPS-provoked BMDM *in vitro* inflammation paradigm was instituted to further substantiate the modulatory influence of Omav upon M1/M2 phenotypic disposition. Flow cytometric interrogation was employed to discern the M1 phenotype (CD86+) (Figure 6B), and the findings disclosed that Omav administration attenuated the M1 polarization of LPS-stimulated BMDMs (Figure 6C). Simultaneously, flow cytometry was used to detect the M2 phenotype (CD206) (Figure 6D), and the results indicated that Omav treatment promoted M2 polarization (Figure 6E).

### 3.6 Omav regulates LPS-induced BMDMs polarization toward the M2 phenotype *in vitro*

**Figure 7:**
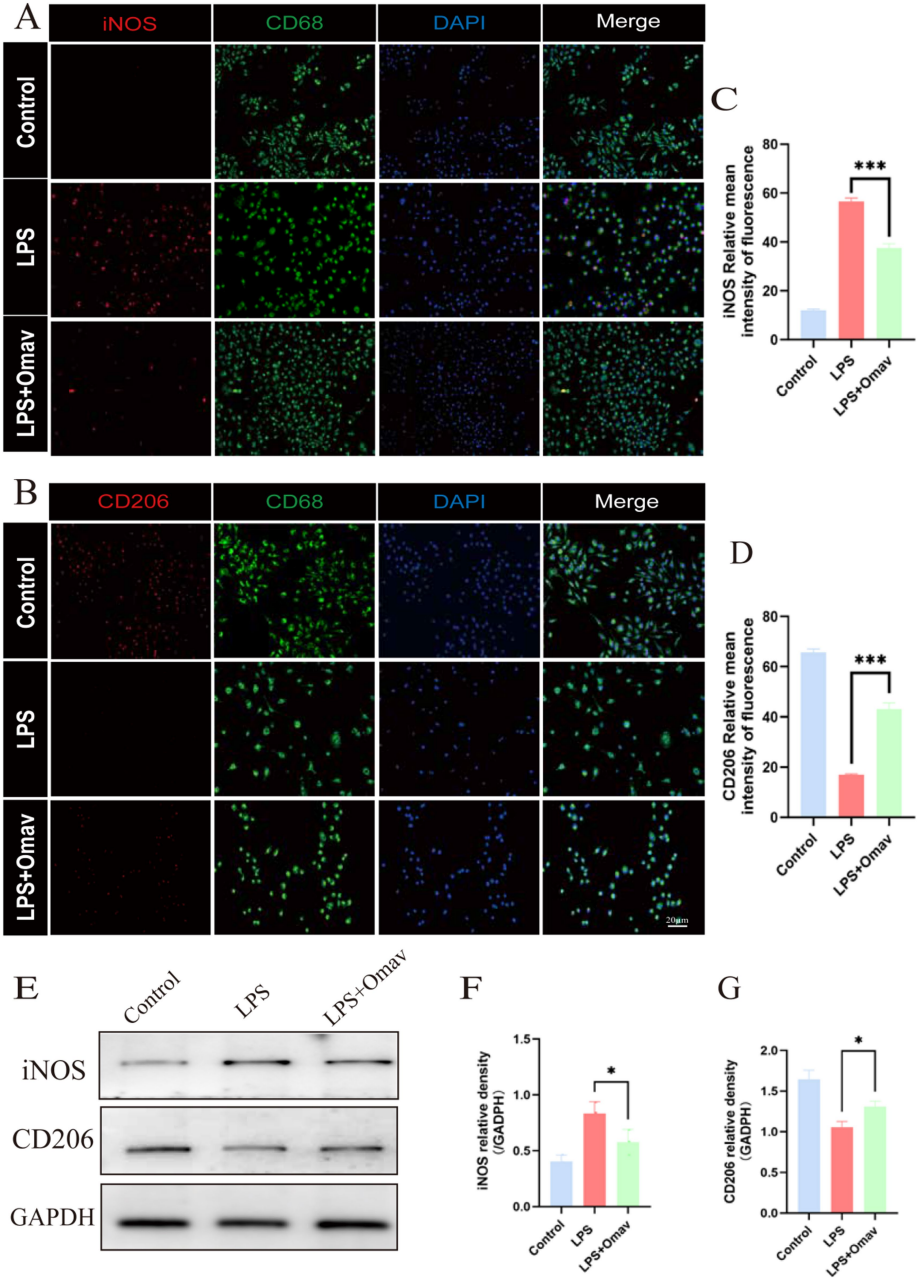
The regulation of Omav on M1/M2 polarization in LPS-stimulated BMDMs. **(A)** Exemplary iNOS (red) and CD68 (green) immunofluorescence images, counterstained with DAPI (blue). Scale bar: 20 μm. **(B)** Representative immunofluorescence micrographs showcasing CD206 (red) and CD68 (green) staining, juxtaposed against DAPI nuclear labeling (blue). Scale bar: 20 μm. **(C)** Image analysis data manifesting the relative mean intensity of iNOS fluorescence (*n* = 3). **(D)** Comparable image analytics reveal relative mean intensity values for CD206 fluorescence (*n* = 3). **(E–G)** Western blot results unveil protein expression profiles and corresponding quantifications of iNOS and CD206 across the distinct experimental cohorts (*n* = 3).

In an effort to further corroborate Omav’s modulatory influence on M1/M2 phenotypes, we resorted to immunofluorescence staining of macrophage identifier CD68 and M1 marker iNOS (Figure 7A). Consequential analyses unveiled a conspicuous decrease in relative average iNOS intensity within the LPS + Omav group (Figure 7C). Subsequently, we stained for macrophage marker CD68 and M2 phenotype marker CD206 (Figure 7B). The results showed that in the LPS + Omav group, the relative average intensity of CD206 significantly increased (Figure 7D). Additionally, we validated this mechanism through western blot (Figure 7E), a comprehensive analysis revealed Omav’s efficacy in suppressing M1 polarization within LPS-stimulated BMDMs, as evinced by a marked decline in iNOS expression levels (Figure 7F). Concurrently, a discernible elevation in CD206 levels served as an indicator of augmented M2 polarization (Figure 7G), substantiating Omav’s propensity to foster an anti-inflammatory macrophage phenotype.

### 3.7 Cross-validation at the overall tissue and macrophage levels: confirmation of key genes

**Figure 8:**
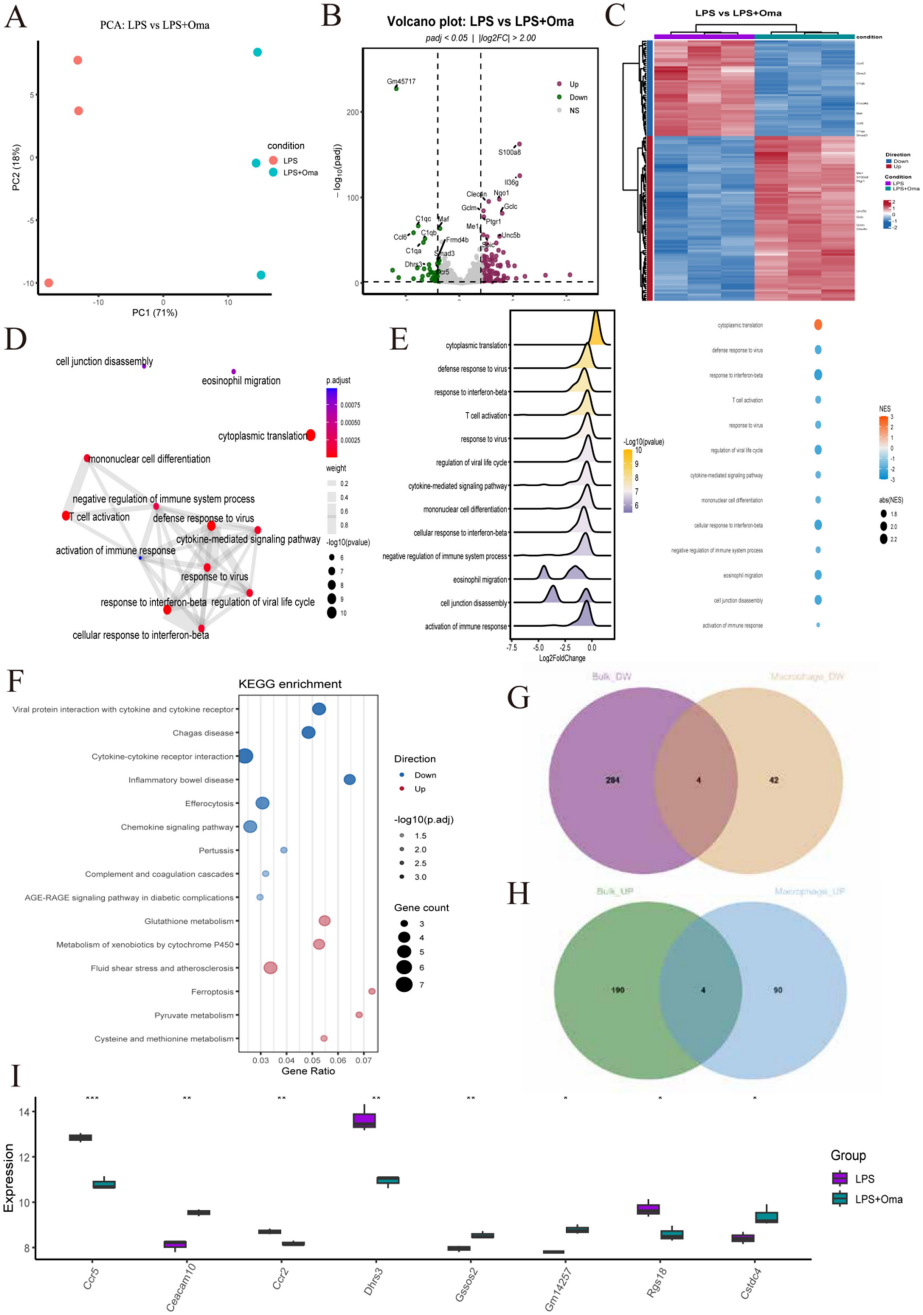
Transcriptome analysis of BMDMs in LPS-induced group and LPS-induced + Omav group. **(A)** Principal-component analysis (PCA): LPS (purple) and LPS + Oma (teal) segregate along PC1, confirming a global Oma effect. **(B)** Volcano plot: 94 up-regulated and 46 down-regulated genes (*p*_adj_ < 0.05, |log₂FC| > 2); key examples labeled. **(C)** Heat-map (top 140 DEGs): columns colored by treatment; left bar marks up- (red) and down- (blue) regulation. **(D)** GO network: enriched biological-process terms clustered by semantic similarity (>0.3). **(E)** GSEA summary: ridgeline and dot plots show pathway direction and NES strength. **(F)** KEGG bubble plot: bubble size = gene count; color = −log₁₀(*p*_adj_); top immune and redox pathways highlighted. **(G,H)** Four genes shared between bulk and macrophage sets for both up- (Ceacam10, Gssos2, Cstdc4, Gm14257) and down-regulated lists (Ceacam10, Dhrs3, Ccr2, Ccr5). **(I)** Box-and-jitter plots compare eight key transcripts—Ccr5, Ceacam10, Ccr2, Dhrs3, Gssos2, Gm14257, Rgs18, and Cstdc4—between the LPS group (purple) and the LPS + Oma group (teal).

To further study the effects of Omav on macrophages in the inflammatory response process after SCI, we conducted Omav drug intervention in the LPS-induced BMDMs *in vitro* inflammation model and analyzed RNA-seq data results. Principal component analysis results indicated clear separation between the LPS group and the LPS + Omav group in gene expression, consistent with the sequencing results of tissue samples. This emphasizes the significant impact of Omav treatment on macrophage inflammatory responses (Figure 8A).

Differential gene volcano plots and heatmaps clearly showed that under LPS-induced inflammatory conditions, several pro-inflammatory and immune activation genes were significantly downregulated after drug treatment (e.g., CCR5, C1q family), while anti-inflammatory or antioxidant protective genes (e.g., Mt1, Gclc, Gclm) were upregulated, indicating that Omav can effectively reduce inflammation caused by M1 macrophages and promote the tissue protection or repair of M2 macrophages. These gene changes reflect the effective regulation of the drug on immune responses and inflammatory processes (Figures 8B,C). GO-BP and GSEA analysis results indicated that the changes in gene expression of macrophages after drug (Omav) treatment were mainly concentrated in immune regulation, interferon signaling pathways, and cytoplasmic translation, suggesting that the regulation of these pathways or biological processes may be one of the important mechanisms by which the drug exerts anti-inflammatory and tissue repair effects (Figures 8D,E). KEGG pathway enrichment analysis results confirmed that Omav treatment significantly downregulated immune-inflammatory-related pathways (e.g., chemokine and cytokine pathways) while upregulating antioxidant and cell protection-related pathways (e.g., glutathione metabolism, ferroptosis pathways), demonstrating that Omav effectively improves the LPS-induced inflammatory state by regulating immune inflammatory responses and antioxidant protective pathways (Figure 8F).

By integrating RNA-seq data at the tissue level and BMDMs level (Figures 8G,H), we ultimately identified eight key target genes (Figure 8I). Among them, four genes were commonly upregulated in both datasets (Ceacam10, Gssos2, Gm14257, Ctsd4), while the other four were commonly downregulated (Ccr5, Ccl2, Rgs18, Dhrs3). The upregulated Ctsd4/CTSD can activate the lysosomal pathway of macrophages/microglia, promoting the clearance of myelin and necrotic cells, thereby reducing secondary injury; while the regulation of genes such as Ccr5 and Ccl2 is closely related to apoptosis, inflammation, and the inhibition of glial scars.

Subsequently, we performed quantitative analysis of CTSD, CCR5, and CCL2 using qRT-PCR. Compared to the SCI group, Omav treatment significantly upregulated the mRNA expression level of the lysosomal enzyme cathepsin D (CTSD) (Figure 7J). Correspondingly, the mRNA expression levels of CCL5 receptor 5 (Ccr5) and C-C chemokine ligand 2 (CCL2) were significantly decreased after Omav treatment compared to the SCI group (Figures 8K,L). The above cross-validation results indicate that Omav treatment significantly weakened the inflammatory response of macrophages, highlighting the central role of inflammation regulation in the pharmacological mechanism of Omav.

## 4 Discussion

SCI constitutes a grave insult to the central nervous system, routinely eventuating in a constellation of motor, sensory, and autonomic disturbances, significantly affecting the patient’s activities of daily living (He et al., 2024; Jiang et al., 2023; Lin et al., 2025). The pathophysiological mechanisms of SCI are complex, involving multiple factors such as neuronal damage, inflammatory responses, and changes in the microenvironment (Wang and Bai, 2024; Kwon et al., 2004; Mortazavi et al., 2015). Although several studies have explored the mechanisms of neural repair after SCI (Baptiste et al., 2009; Pang et al., 2025), there are currently no effective interventions that can achieve complete functional recovery. Capitalizing on extant knowledge, our investigations unveil Omav’s ability to foster post-SCI functional restoration by orchestrating macrophage polarization dynamics and curtailing inflammatory cascades. These revelations illuminate Omav’s anti-inflammatory modus operandi within SCI therapeutics, broadening the horizon of potential interventions for this debilitating condition. As such, our findings embody a promising therapeutic approach, providing insights for more efficacious SCI treatment strategies.

After SCI, Omav plays a positive role in alleviating inflammation (Reisman et al., 2020; Jiang et al., 2022; Jian et al., 2022). However, the role of Omav in the progression of SCI remains unclear. In this study, we found that Omav exhibits significant anti-inflammatory effects and potential repair mechanisms by regulating macrophage M1/M2 polarization and corresponding signaling pathways. Our research shows that Omav can significantly modulate macrophage M1/M2 polarization, tempering M1 macrophage prevalence while augmenting the M2 counterpart population. This strategic orchestration of macrophage phenotypes tames inflammatory surges and cultivates a favorable milieu for tissue regeneration. This study employs various experimental methods, including RNA-seq analysis, to reveal the anti-inflammatory mechanisms of Omav after SCI and its impact on functional recovery. Preliminary results indicate that Omav can significantly alter the gene expression profile after SCI, regulate macrophage M1/M2 polarization, and promote tissue repair. These findings provide a theoretical basis for the clinical application of Omav as a potential therapeutic drug. Moreover, we identified 482 DEGs after Omav treatment, of which 287 genes are downregulated and 195 genes are upregulated. These DEGs suggest that Omav plays a key role in regulating immune responses and promoting tissue repair, particularly by downregulating genes associated with inflammation and apoptosis and upregulating genes related to tissue repair. Functional module analysis of these DEGs revealed their important roles in biological processes such as nerve regeneration, immune regulation, and cell apoptosis, thereby providing a molecular mechanistic basis for the application of Omav in SCI treatment. In addition, the effect of Omav on the immune response has also been confirmed in this study. GO and GSEA showed that Omav significantly inhibited the activation of immune response-related and inflammation-related pathways. Meanwhile, it mildly activated tissue repair-related pathways. This finding suggested that Omav not only suppresses the inflammatory response after SCI, but also enhances functional recovery by promoting tissue repair. These dual actions—immune suppression and tissue repair promotion—may provide new theoretical support for Omav as a novel therapeutic drug, particularly in the clinical application of nerve regeneration and functional recovery.

This study has several limitations. First, the research mainly focuses on macrophage polarization and inflammatory response, but does not deeply explore the core molecular link between Nrf2 activation and direct M2 polarization. Second, we have established a contusion model of SCI and have not addressed other SCI animal injury models.

## 5 Conclusion

In summary, Omav alleviates the inflammatory response after SCI and promotes functional recovery by regulating macrophage M1/M2 polarization and related signaling pathways. Specifically, studies have shown that Omav significantly inhibits immune responses and inflammatory pathways while activating tissue repair pathways. It also promotes M2 macrophage polarization and reduces the M1 phenotype, thereby providing a new therapeutic strategy for SCI.

## Data Availability

The datasets presented in this study can be found in online repositories. The names of the repository/repositories and accession number(s) can be found in the article/Supplementary material.
