# Supplementary material for: Omaveloxolone promotes functional recovery of spinal cord injury by reducing inflammatory response and regulating macrophage polarization
Source: Front Mol Neurosci. 2026 Jan 12;18:1737798. doi: 10.3389/fnmol.2025.1737798 (PMC12833412; doi:10.3389/fnmol.2025.1737798)
Supplement: Supplementary file 1 [file Data_Sheet_1.PDF]

**Supplementary Figure 1**

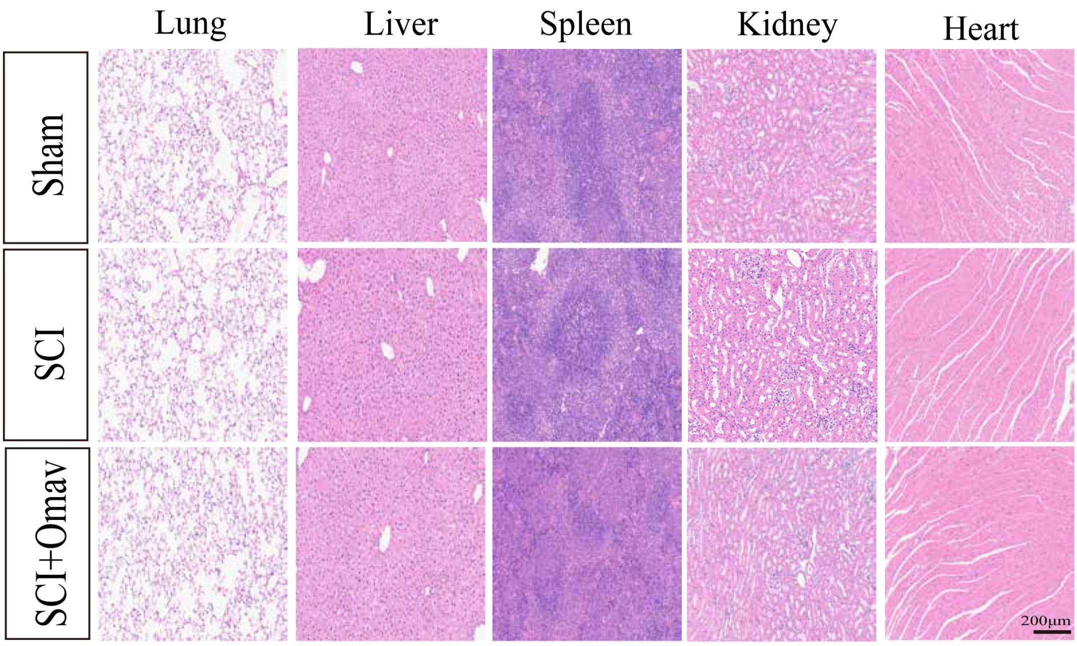


**Fig. S1. Liver, kidney, lung, heart, and spleen tissue H&E staining from Sham controls, PBS-treated SCI controls, and SCI mice treated with Omav (n=3).**
